# Peptide design to control protein–protein interactions

**DOI:** 10.1039/d4cs00243a

**Published:** 2025-01-16

**Authors:** Suzanne P. van Wier, Andrew M. Beekman

**Affiliations:** a School of Chemistry, Pharmacy & Pharmacology, University of East Anglia Norwich Research Park Norwich NR4 7TJ UK a.beekman@uea.ac.uk

## Abstract

Targeting of protein–protein interactions has become of huge interest in every aspect of medicinal and biological sciences. The control of protein interactions selectively offers the opportunity to control biological processes while limiting off target effects. This interest has massively increased with the development of cryo-EM and protein structure prediction with tools such as RosettaFold and AlphaFold. When designing molecules to control protein interactions, either inhibition or stabilisation, a starting point is commonly peptide design. This tutorial review describes that process, highlighting the selection of an initial sequence with and without structural information. Subsequently, methods for how the sequence can be analysed for key residues and how this information can be used to optimise the ligand efficiency are highlighted. Finally a discussion on how peptides can be further modified to increase their affinity and cell permeability, improving their drug-like properties, is presented.

## Abstract 2

Targeting of protein–protein interactions has become of huge interest in every aspect of medicinal and biological sciences.

## Graphical abstract

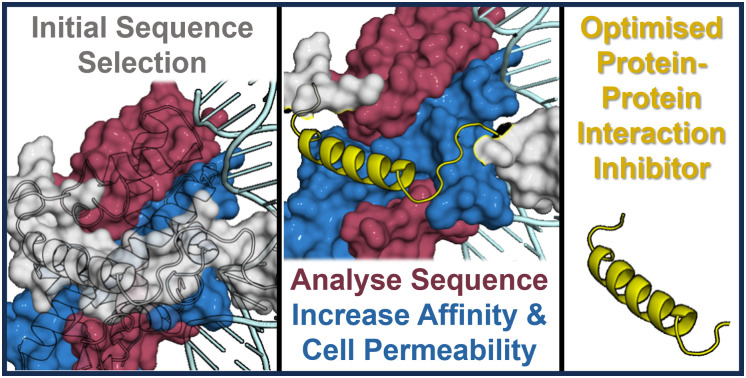

**Key learning points**

1. Proven methods for identifying key interfaces in protein interactions.

2. How to identify key sequences for peptide–protein binding.

3. How to design probes for protein interactions where structure is available.

4. How to design probes for protein interactions where structure is unknown.

5. How to modify peptides to improve their value as chemical tools.

## 1. Introduction

Protein–protein interactions (PPIs) modulate many biological processes, and dysregulation of these interactions leads to aberrant biology. The selective control of protein interactions offers the opportunity to control biological processes in all kingdoms of life. For example, control of targets in cancer, bacterial infection and immunity, and crop protection and food security.^1,2^ However, targeting PPIs is challenging in comparison to classical drug targets, such as enzymes, receptors and ion channels, as PPIs often take place across large, flat surfaces (1500–3000 Å) compared to the smaller, well-defined binding pockets of classical targets (300–1000 Å).^3^ As such, PPIs have been classed as ‘undruggable’, leaving many PPIs still largely unexplored in drug discovery and it is thus important to find new methods and improve existing techniques to target PPIs.

**Fig. 1:**
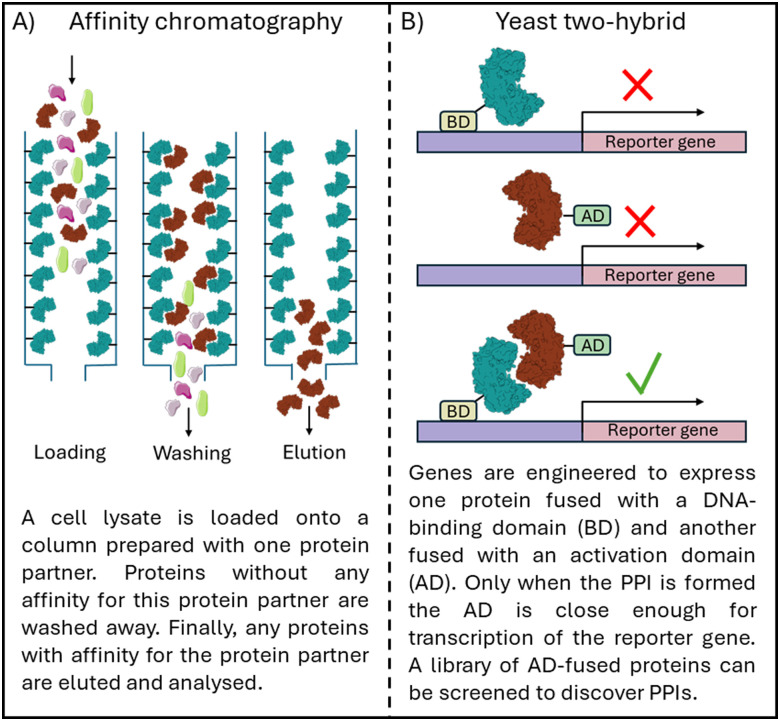
Schematic overview of (A) affinity chromatography to identify PPIs and (B) a yeast two-hybrid screen to confirm PPI formation.

Proteins and their interacting partners can be identified *in vitro* with methods such as affinity chromatography using immobilised protein (Fig. 1A), and co-immunoprecipitation, a technique in which PPIs are captured from the cell lysate.^4,5^*In vivo* yeast two-hybrid screens can confirm PPIs in which the protein complex is required for the transcription of reporter genes, visualised with cell growth or colour change (Fig. 1B).^6,7^ To understand the importance of the discovered PPIs, silencing RNA or gene knockout technology can inhibit the expression of one of the protein partners and the resulting cellular effect observed.^8,9^ However, protein knockout abolishes all interactions for the targeted protein, and as such, modulators able to control individual protein interactions are vital chemical tools for understanding biology. With the progress in cryo-EM for protein complexes and machine learning for protein multimer structure prediction, such as Alphafold, tools for understanding protein–protein interactions are increasingly democratized.^10,11^ These tools and the information they present allow for greater understanding of PPIs in all kingdoms of life, and therefore greater control.

PPIs are commonly modulated using antibodies, peptides, and small molecules. Humanised antibodies are highly selective and effective, exemplified in targeting the PD-1/PD-L1 interaction in the human immune system, for which multiple antibody treatments have been approved.^12^ However, negatives to antibody approaches are their high production cost, low tissue penetration and adverse immune responses.^13^ Their use has been limited to extracellular targets, although development of intrabodies, intracellularly active antibodies, is ongoing.^13,14^

Peptides provide an alternative to antibodies with many benefits, including simpler production at lower cost, while providing specific targeting of interactions, unlike small molecules.

**Fig. 2:**
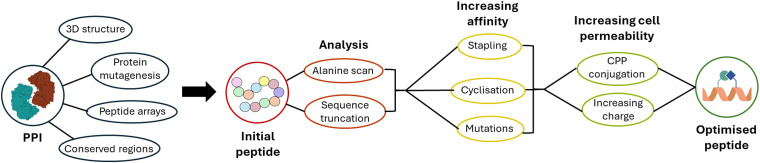
Schematic overview of the methods discussed in this review, from selecting the initial peptide sequence, to analysing this, increasing affinity for the target protein as well as cell permeability, leading to an optimised peptide.

Identifying peptides from the secondary structures responsible for the targeted PPI promises efficacy and selectivity, and a conceptually simple starting point for modulator development. Compared to other peptide discovery techniques such a phage and mRNA display, it also requires less specialist knowledge and equipment. This review describes approaches for the selection of an initial peptide sequence, and the processes for optimising the peptide for affinity and cell permeability (Fig. 2).

### 1.1 Biophysical/biochemical assays

**Fig. 3:**
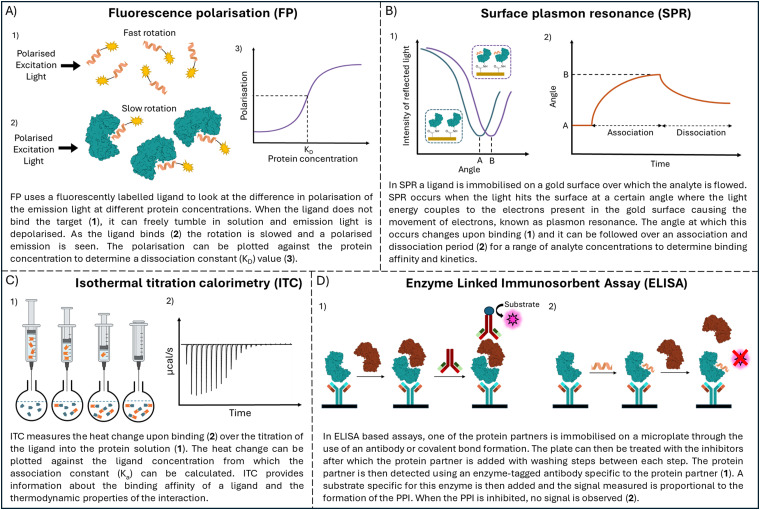
Schematic overview techniques used to analyse peptide–protein interaction with (A) fluorescence polarisation (FP), (B) surface plasmon resonance (SPR), (C) isothermal titration calorimetry (ITC) and (D) enzyme linked immunosorbent assay (ELISA).

To understand how peptide sequences are discovered, it is valuable to first appreciate techniques used to detect and measure peptide–protein interactions. Once lead molecules have been synthesised, they must be evaluated against the target protein. *In vitro* biophysical assays are routinely used in early drug discovery to investigate protein–ligand interactions. For the inhibition of PPIs, the ability of the ligand to disrupt the PPI formation is also of interest. *In vitro* assays to examine these properties are a useful tool in the development of PPI inhibitors (Fig. 3).

Fluorescence polarisation (FP), microscale thermophoresis (MST), and surface plasmon resonance (SPR) can provide binding affinities, whereas differential scanning fluorimetry DSF is better used as a relative comparison tool between compounds. SPR provides information on the kinetics of the interaction. Isothermal titration calorimetry (ITC) can provide detailed information on kinetics and binding affinity but is poorly suited for high throughput application. However, it is the only technique that is label-free and in solution providing the most accurate information about the interaction. FP and MST rely on the use of a fluorescent label on the ligand or protein and DSF requires the addition of a fluorescent dye. Surface plasmon resonance (SPR) and enzyme linked immunosorbent assay (ELISA) requires an analyte to be immobilised, which could interfere with the binding.

Assays based on resonance energy transfer such as Förster resonance energy transfer (FRET) and homogeneous time resolved fluorescence (HTRF), can take place with all components in solution, allowing evaluation of PPI formation.

## 2. Selection of an initial peptide sequence

If structural information is available, the choice of peptide sequence starting point can be made by analysing the binding interface. However, without this structural information or knowledge of which part of the interaction is most important, methods including protein mutagenesis, sequence conservation analysis and peptide arrays can be used.

### 2.1 Selection of peptides through structural information

When the structure of a protein is known a direct approach to designing a peptide inhibitor is through analysing the structure and identifying the interaction face of the protein partner. The interacting section can be taken from this as a starting point before further optimisations take place.

**Fig. 4:**
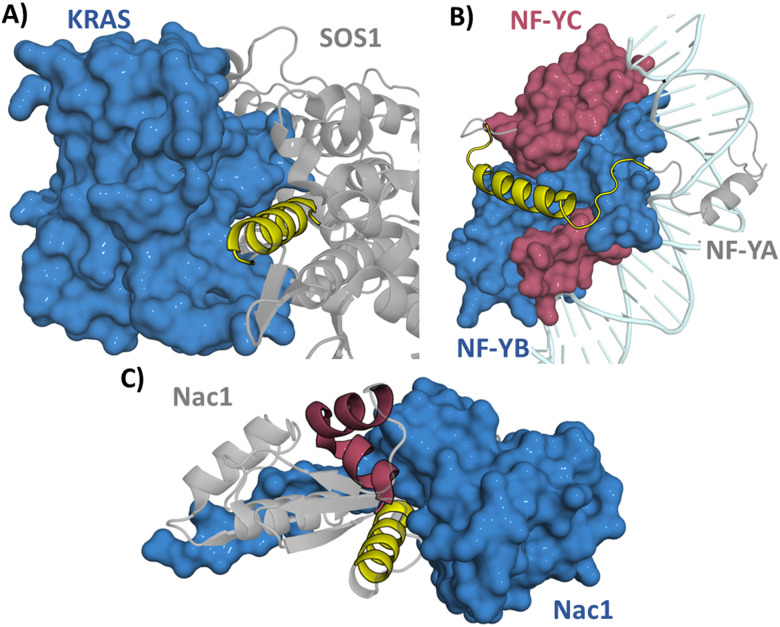
(A) Structure of SOS1 (grey)/KRAS (blue) (PDB: 1NVU) with the sequence _929_FFGIYLTNILKTEEGN_944_ (1) in yellow, (B) structure of NFY trimer (PDB: 4AWL). NF-YA (grey) with peptide sequence _267_VNAKQYHRILKRRQARAKLEAEGKIPKER_295_ (2) (yellow), NF-YB (blue) and NF-YC (burgundy) and (C) structure of the Nac1 dimer (PDB: 3GA1) with section _12_FGNSILECLNEQR_24_ (3) in yellow and _44_HRAVLAASSSYFRDLFN_60_ (4) in burgundy.

If the interaction is mediated through an α-helix the whole helix can be used as a starting point for the interaction. For example, Kirsten rat sarcoma viral oncogene homolog (KRAS) is a small GTPase which is involved in cell survival and proliferation. KRAS mutations are found in cancers and lead to decreased GTPase activity which in turn leads to increased RAS pathway signalling.^15^ KRAS is negatively regulated by son of sevenless 1 (SOS1) and therefore the inhibition of the KRAS/SOS1 PPI can increase GTPase activity.^16^ The SOS1 derived section _929_FFGIYLTNILKTEEGN_944_ (1) forming an α-helix (Fig. 4A) was identified from the crystal structure and used as the starting point for inhibitor development.^17,18^ Depending on the interaction site a region around the α-helix may also be included if the structure suggests it is part of the interacting sequence, exemplified in targeting the nuclear transcription factor Y (NF-Y) trimer formation. NF-Y activates the genes associated with cell cycle regulation and DNA repair with overexpression of NF-Y found in cancer.^19^ Residues _267_VNAKQYHRILKRRQARAKLEAE-GKIPKER_295_ (2) were taken from NF-YA encompassing an α-helical section which continues into an interacting disordered section (Fig. 4B). This section was shown to bind to NF-YB/C dimer (*K*_D_ = 0.7 μM) in a fluorescence polarisation (FP) assay. This sequence was used as a starting point allowing exploration of truncated sequences to determine if the disordered section is crucial for the interaction (see Section 3.2).^20^ This approach was also taken in targeting the RbAp48/MTA1 interaction which consists of both an α-helical and disordered section.^21,22^ The approach of deriving a peptide starting point from the sequence has now been taken for targeting many α-helix mediated PPIs, such as Axin/β-catenin,^23^ BCL9/β-catenin,^24^ Ca_v_/Ca_v_β,^25^ Cullin3/KCTD11^26^ and SARS-CoV-2 spike/ACE2.^27^

The protein structure may also reveal more than one potential interaction site between the protein and its partner. Nucleus accumbens-associated protein 1 (Nac1) is a repressor protein that mediates the interactions between transcription factors with an essential role in carcinoma tumour growth.^28^ In targeting Nac1 homodimerization two separate sections were explored as initial sequences: _12_FGNSILECLNEQR_24_ (3) and _44_HRAVLAASSSYFRDLFN_60_ (4) with 3 showing weak binding in an FP assay (*K*_D_ = 360 μM) and 4 showing no binding (Fig. 4C). Regardless, both sections were further explored to see whether their affinity could be improved.^29^

If the target protein has multiple protein partners, these could all be explored as potential starting points. The misregulations of Ras-related in brain (Rab) proteins are implicated in neurodegenerative diseases and cancer. In targeting these proteins crystal structures with multiple different protein partners were explored, identifying nine sequences.^30,31^ These were tested against a set of seven Rab proteins in an FP assay.

**Fig. 5:**
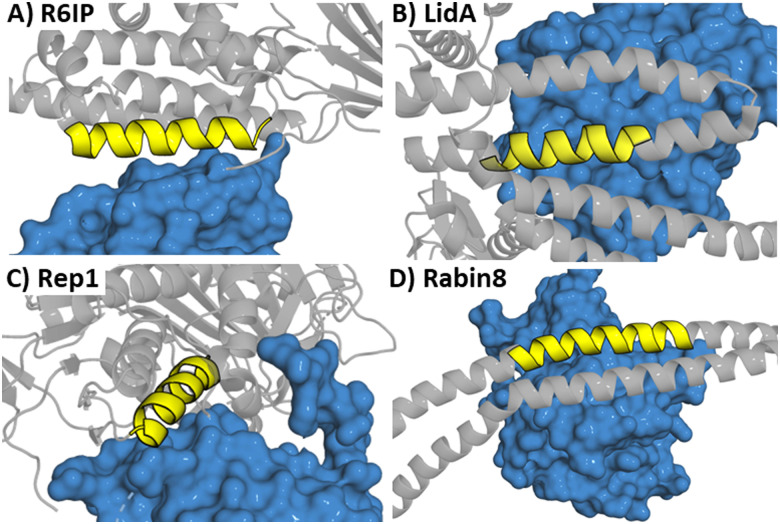
Structures of a Rab protein in complex with (A) R6IP (PDB: 3CWZ), (B) LidA (PDB: 3TNF), (C) Rep1 (PDB: 1VG0) and (D) Rabin8 (PDB: 4LHX) highlighting the peptide starting sequence in yellow.

**Fig. 6:**
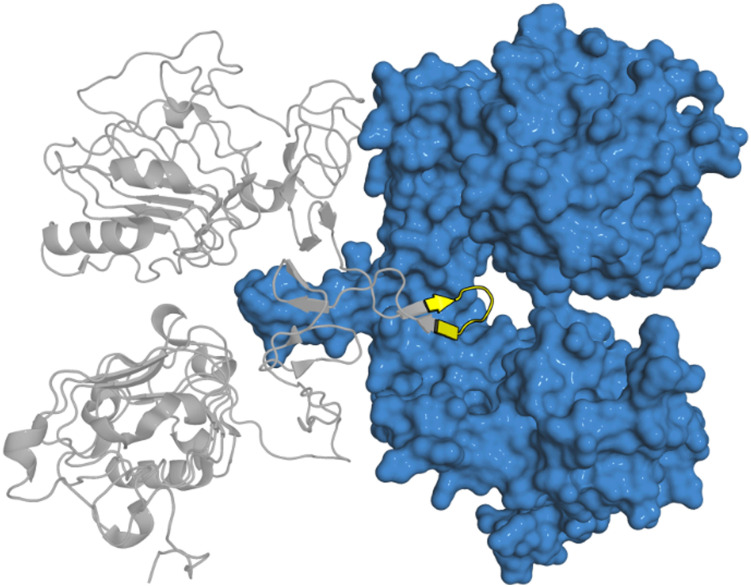
Structure of EGFR dimer (PDB: 1IVO) with β-hairpin starting peptide sequence _269_YNPTTYQM_278_ (5) highlighted in yellow.

Four peptides (derived from R6IP, LidA, REP1 and Rabin8, Fig. 5) with low micromolar binding affinities were further explored.^31^ Using structural information is not limited to α-helical motifs with the same approach deployed for β-hairpin sections. This has been applied to targeting the epidermal growth factor receptor (EGFR) which is overexpressed in cancer.^32^ EGFR forms an asymmetric dimer and disruption of the dimerization could inactivate the kinase, which was explored by mimicking the EGFR dimerization arm residues _269_YNPTTYQM_278_ (5) which form a β-hairpin (Fig. 6).^33^

The continued democratization of cryo-EM will undoubtedly increase the experimental data for protein–protein interactions.

Similarly, the continued progress in protein structure prediction, including protein multimer, protein–DNA, and protein–RNA will provide new structural insight for identifying initial peptides for development. To date, this has been throttled by the poor accuracy of structure prediction tools^34–36^ for peptide–protein interactions, limiting confidence in peptide design. But recent reports into virtual array development^37^ and improvement in biomolecular structure prediction^38^ will continue to advance this area.

### 2.2 Using site-directed protein mutagenesis

Protein mutagenesis is a technique in which a mutation is introduced into the genes encoding for the desired protein. This can be used to analyse which residues within the protein are most important for the interaction. For example, this can be used for the expression of alanine mutants. The amino acid alanine has a methyl group as its side chain substituent. Compared to other amino acids, which have either larger hydrophobic groups in its place or polar or charged side chains, the methyl is not able to form strong interactions with the target protein. The expression of alanine mutants that result in a loss of binding affinity suggest a hot spot residue, the small subset of residues that contribute most of the binding energy in a PPI.^39,40^ Hot spots are defined as residues which upon mutation to alanine lead to a loss of 2.0 kcal mol^−1^ or more in binding free energy.^41^ Protein mutagenesis is a useful tool to determine the best starting point when the PPI is comprised of multiple binding interfaces. This approach can be combined with computational prediction to select which residues to mutate minimising the number of proteins generated.

**Fig. 7:**
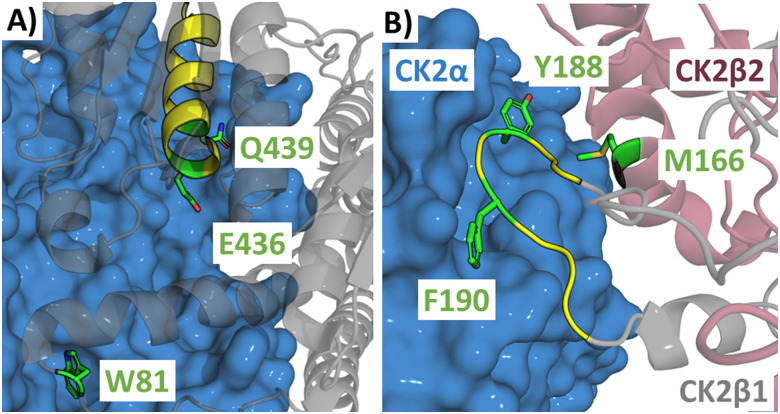
(A) Structure of the *Leishmania infantum* TryR dimer (PDB: 2JK6) highlighting residues W81, E436 and Q439 in green with the starting peptide section _435_PEIIQSVGICMKM_447_ (6) highlighted in yellow and (B) structure of CK2α (blue) in complex with CK2β1 (grey) and CK2β2 (purple) (PDB: 1JWH) highlighting residues M166, Y188 and F190 in green with the section _186_RLYGFKIH_193_ (8) used as the starting point in yellow.

This approach was applied in targeting *Leishmania infantum*, a parasitic disease causing visceral leishmaniasis. Trypanothione reductase (TryR) maintains the intracellular redox state and is essential for parasitic activity.^42^ The homodimer interface of TryR was explored computationally to identify potential hot spots by examining the burial of solvent-accessible surface area, the van der Waals and electrostatic contributions of each residue to the interaction. Three mutant proteins (W81A, E436A and Q439A, Fig. 7A) were expressed and analysed using native polyacrylamide gel electrophoresis (PAGE), a technique used to separate proteins and protein complexes by size, showing that only the E436A mutation decreased dimer formation.^43^ This residue is part of an α-helix (_435_PEIIQSVGICMKM_447_ (6)) chosen as the starting point for peptide design. The cysteine was replaced with serine to avoid disulfide bond formation leading to peptide 7 (Ac-PEIIQSVGISMKM-NH_2_), which was able to disrupt the dimer formation in an Enzyme Linked Immunosorbent Assay (ELISA) with an IC_50_ of 40.8 μM.^43^

Casein kinase 2 (CK2) is a protein kinase with functions in cell proliferation and apoptosis suppression. Overexpression of CK2 has been found in tumours, making it an attractive cancer target.^44^ CK2 consists of two catalytic (α) and two regulatory (β) subunits and as an inhibitory strategy the CK2α/CK2β PPI was targeted. Three residues of CK2β (M166, Y188 and F190, Fig. 7B) were identified as pointing directly towards CK2α with Y188 and F190 found together on one of the CK2β subunits and M166 found on the other CK2β subunit. A single F190A mutant showed significantly reduced binding and a double (F190A + Y188A) and triple (F190A + Y188A + M166A) mutants abolished binding as observed by surface plasmon resonance (SPR).^45^ Y188 and F190 are part of the same β-hairpin loop (_186_RLYGFKIH_193_ (8)) which was taken as the starting point for the design of cyclic peptides. The sequence was not tested as a linear peptide, but a disulfide cyclised 13-mer peptide GC*RLYGFKIHGC*G (9, *cyclised residues) demonstrated a of *K*_D_ = 1.75 μM by Isothermal titration calorimetry (ITC).^45,46^

Site-directed mutagenesis can also confirm the importance of mutations found in disease, guiding the design of peptides. Phosphatidylinositol 3-kinase α (PI3Kα) is involved in cell proliferation forming interactions with adapter proteins such as insulin receptor substrate 1 (IRS1) in the presence of growth factor stimulation, stimulating the catalytic activity of PI3Kα.^47^ The two most common mutations found in cancer (E545K and H1047R) were explored experimentally identifying IRS1 as a binder of E545K mutant protein but not of H1074R mutant protein in pull-down experiments.^48^ It was also shown that the interaction of E545K mutant PI3Kα with IRS1 is required for the growth of colon cancer cells *in vivo*. A 30-residue section consisting of two α-helices around E545 (_528_EQLKAISTRDPLSEITEQEKDFLWSHRHYC_558_ (10)) was chosen for peptide design, which inhibited the PPI formation in cell lysate as investigated by immunoprecipitation. Following this, each helix was explored separately giving the N-terminal 21-mer peptide _528_EQLKAISTRDPLSEITEQEKD_549_ (11) and the C-terminal 18-mer peptide _541_SEITEQEKDFLWSHRHYC_558_ (12) with only the C-terminal peptide 12 able to disrupt the PPI.^48^

**Fig. 8:**
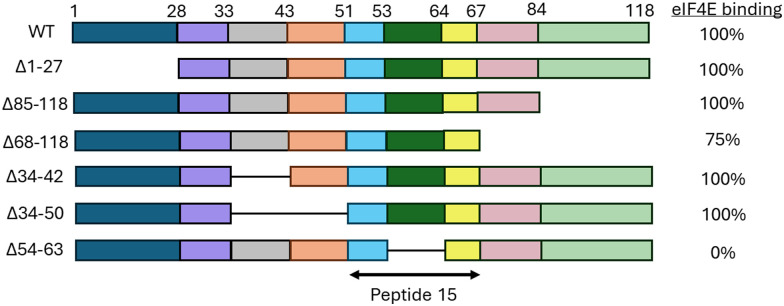
Sequences created in scanning deletion mutagenesis of 4E-BP1.

Deletion scanning mutagenesis can be used to investigate the binding regions of a PPI through the expression of mutant variants, where parts of the protein have been removed. Eukaryotic translation initiation factor 4E (eIF4E) plays a role in the initiation of translation with overexpression inducing tumorigenesis.^49^ eIF4E is negatively regulated by eIF4E-binding protein 1 (4E-BP1) through competition with eIF4G binding which is required for the formation of the active complex needed for translation.^50^ Peptides mimicking 4E-BP1 were explored to inhibit translation through stopping the eIF4E-eIF4G PPI formation. Mutagenesis was used to remove the genes encoding for sections across residues 1–118 of the 4E-BP1 protein (Fig. 8). Only the mutant without the 54–63 section of 4E-BP1 did not bind eIIF4E, showing this section to be crucial for binding. Therefore, it was chosen to start the peptide design from the longer _51_RIIYDRKFLMECRNSPV_67_ (13) section encompassing residues 54–63. This peptide bound eIF4E with a *K*_D_ of 50 nM as measured by ITC.^51^

### 2.3 Peptides spanning protein sequence

If the interacting site of the PPI is not known peptides probing the entire protein sequence can be generated. The synthesis and purification of many peptides can be time-consuming using solid phase peptide synthesis. Instead, techniques such as peptide arrays have been designed in which many peptides can be probed at the same time.

#### 2.3.1 Peptide arrays

**Fig. 9:**
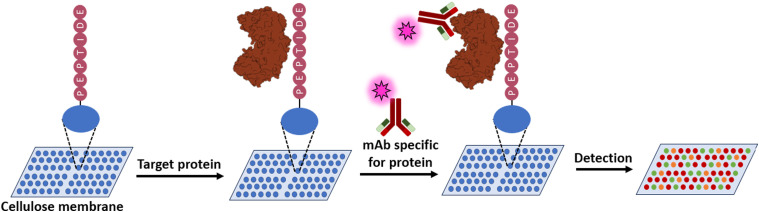
Schematic overview of a peptide array.

In a peptide array peptides are synthesised on a cellulose membrane.^52^ This membrane can be incubated with the target protein, after which it can be washed to remove any unbound protein. Any bound protein to the peptide can then be detected with an antibody (Fig. 9). This approach allows a quick screening of overlapping peptide sequences to identify which section of the protein is able to bind the target protein.^53^ This technique was applied to find new antiviral peptides for human immunodeficiency virus (HIV) to target the PPI between human apolipoprotein-B mRNA-editing catalytic polypeptide-like 3G (A3G) and HIV-1 Virion infectivity factor (Vif) protein which leads to the degradation of A3G stopping the anti-viral activity of A3G.^54^ The inhibition of this PPI was thought to rescue the activity of A3G. A peptide array of both A3G and Vif was designed with peptides of approximately 15 residues in length with 7 or 8 residues of overlap. This led to the identification of A3G derived peptide _211_WVRGRHETYLCYEVE_225_ (14) able to inhibit HIV-1 propagation.^55^

The same approach was also taken in targeting the BH3-interacting domain death agonist (BID) and mitochondrial carrier homologue 2 (MTCH2) PPI, an interaction involved in apoptosis. Two peptides (_59_WTDGNRSSHSRLGRIE_73_ (15) and _111_WLQLRNTSRSEEDRNR_125_ (16)) were found to induce near complete cell death at 50 μM in osteosarcoma cells as penetratin, a cell-penetrating peptide, conjugates.^56^

#### 2.3.2 Solid phase peptide synthesis approaches

Although potentially time-consuming a similar approach as a peptide array can be performed with solid phase peptide synthesis. It can be helpful to narrow down the number of peptides required by focussing on the structured α-helices and β-sheets found within a protein. This was demonstrated when targeting HIV-1 integrase (IN). HIV-1 IN is required for viral replication and therefore of interest as an HIV drug target. HIV-1 IN forms multimers and interacts with proteins such as lens epithelium-derived growth factor (LEDGF) and DNA.^57^ 16 peptides spanning the protein between residues 10–267 were created to probe the α-helices and β-sheets found within the N-terminus, catalytic core and C-terminus and they were tested for HIV-1 integrase Inhibition.^58^ The designed peptides were synthesised using solid-phase peptide synthesis and then tested for HIV integrase catalytic activity. This resulted in the identification of two peptide sequences: _97_TAYFLLKLAGRW_108_ (17) and _129_ACWWAGIKQEF_139_ (18) which showed inhibition of integration (also known as strand transfer) with an IC_50_ of 2.7 μM and 56 μM respectively with further work needed to determine their mode of action.^58^

It might not be necessary to probe the entire protein as was the case in targeting the β-barrel assembly machine (Bam), present in the outer membrane of Gram-negative bacteria. Bam contains the two essential proteins BamA and BamD. Here it was first determined through pull-down experiments (an experiment in which a protein is immobilised, and it is evaluated whether its protein partner can be captured from cell lysate) of BamD with urea-denatured fragments of BamA, that only the C-terminal residues 715–810 were able to bind BamD. Therefore five overlapping sequences spanning this region were designed with only the peptide spanning residues 765–779 effective at inhibiting the assembly of Bam.^59^

### 2.4 Conserved regions across proteins or species

Many proteins are conserved across species or across groups of proteins with similar functions, which can indicate the importance of these regions, and suggest an initial sequence for binding and inhibiting target PPIs.

**Fig. 10:**
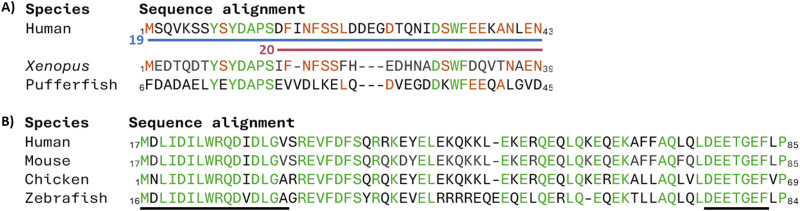
(A) Sequence alignment of human, *Xenopus*, and pufferfish TPX2 sequence highlighting in green the residues conserved across all three species and in orange the residues conserved across two species. Peptide 19 is highlighted in blue and peptide 20 in maroon. (B) Sequence alignment of human, mouse, chicken, and zebrafish Nrf2 sequence highlighting in green the residues conserved across all four species and underlined the sections 21 and 22.

Targeting protein for Xklp2 (TPX2) is a spindle assembly factor which is required during cell mitosis. TPX2 localises Aurora-A, an essential mitotic kinase to spindle microtubules which has been highlighted as an oncogene.^60^ Residue conservation across human, *Xenopus* and pufferfish TPX2 is seen across residues 1–43 (19) (Fig. 10A) and immunoprecipitation and pull-down experiments showed the interaction of 19 with Aurora-A whilst also showing that the shorter 15–43 section (20) was not able to do so.^61^

Nuclear factor erythroid 2-related factor 2 (Nrf2) is a transcription factor leading to the transcription of cytoprotective genes. Kelch-like ECH-associated protein 1 (Keap1) is a negative regulator of Nrf2 tagging Nrf2 for degradation by ubiquitination. To target the Nrf2/Keap1 PPI the human sequence of Nrf2 was compared to that in mouse, chicken, zebrafish, and drosophila with two highly conserved regions found: residues 17–32 (21) and residues 77–82 (22) containing an DEETGE motif (Fig. 10B). Through ITC measurements of mutant proteins missing either section, it was shown that the binding affinity of 22 was higher than 21.^62^ Therefore a 16-mer peptide (_69_AFFAQLQLDEETGEFL_84_ (23)) around the human _77_DEETGE_82_ motif was used as an initial sequence for inhibitor development, binding Keap1 with a *K*_D_ of 20 nM.^62,63^

Similar approaches have been deployed for DNA polymerases across eubacteria and archaea, leading to the identification of the QL[S/D]LF consensus sequence with further work based on this sequence discussed in multiple reviews.^1,2,64,65^ Similarly, the control of plant ethylene responses was achieved by investigating a conserved nuclear localization signal sequence found in *Arabidopsis* ethylene regulator ethylene insensitive-2 (EIN2).^66–69^

## 3. Analysing the sequence

Once an initial sequence has been identified, it is important to understand the importance of each of the amino acids within the peptide. This can be explored through truncation studies as well as by performing an alanine scan. In the same way as discussed for proteins in Section 2.2, an alanine scan provides information about the contribution of each residue in the sequence to the overall binding affinity of the peptide. Truncation studies can be used to explore a series of sequentially shortened peptides, to understand which section of the sequence is at the core of the binding interface. Together, these techniques provide further information about the sequence, which can then be used to increase the binding affinity (as discussed in Section 4).

### 3.1 Alanine scan

An alanine scan can be used to highlight the hot spot residues within a peptide sequence. In an alanine scan, residues within the sequence are sequentially replaced with alanine and the binding affinity is analysed.

Inducible nitric oxide (NO) synthase (iNOS) produces NO which is required for the intracellular killing of pathogens. iNOS is negatively regulated by SPRY domain-containing SOCS (suppressor of cytokine signalling) box protein 2 (SPSB2) and the linear peptide Ac-KEEKDINNNVKKT-NH_2_ (24) derived from iNOS was shown to bind SPSB2 with a *K*_D_ of 13.3 nM (ITC). Through an alanine scan the most important residues in the sequence for binding were identified in the section _23_DINNN_27_.^70,71^

Disruptor of telomeric silencing 1-like (DOT1L) catalyses the methylation of histone H3 at lysine 79 and has been implicated in leukaemia.^72^ In up to 10% of acute leukaemia cases the mixed lineage leukaemia (MLL) protein is fused to a partner protein such as AF9. The PPI of AF9 with DOT1L recruits DOT1L to AF9 targeted genes increasing their methylation therefore increasing their expression leading to leukaemia. An alanine scan was performed of peptide _865_LPISIPLSTV_874_ (25) (IC_50_ = 0.49 μM by competitive SPR) derived from DOT1L showing that three C-terminal residues were not essential for binding. Therefore it was possible to generate the shortened 7-mer peptide _879_LPVSIPL_886_ (26) with only a small loss in binding affinity (IC_50_ = 3.9 μM).^73^

An alanine scan may also lead to the discovery of a peptide with enhanced binding affinity. An alanine scan of Ac-DEETGEF-OH (27) (IC_50_ = 5.39 μM), a shortened version of the Nrf2 derived peptide 23, found that an E77A mutation increased binding (IC_50_ = 0.730 μM) nearly 10-fold.^74^ YAP residues _86_MRLRKLPDSFFKPPE_100_ (28) form a twisted-coil with R87 and F96 forming a cation–π interaction. The section _81_PQTVP_85_ (29) was previously reported to be essential to binding and therefore the linear peptide _81_PQTVPMRLRKLPDSFFKPPE_100_ (30) was chosen as the starting point.^75^30 gave an IC_50_ of 49 μM by competitive SPR and an alanine scan of 30 revealed that a D93A mutation, giving _81_PQTVPMRLRKLP**A**SFFKPPE_100_ (31) increased binding (IC_50_ of 25 μM).^76^

Alternatively, an alanine scan can highlight unimportant residues which may then later be used to further improve the peptide such as through the introduction of mutations (see Section 4.1) or by peptide stapling (see Section 4.3).^77^

### 3.2 Sequence truncation

Shortening the peptide sequence to contain only the essential residues for the interaction improves ligand efficiency and simplifies synthesis.

Truncation of the sequence derived from the structure of NF-YA (residues 267–295 (2)) was explored (Table 1). Six shortened peptides were generated, first removing residues from the C-terminus until no longer tolerated followed by shortening of the N-terminus until no longer tolerated (Table 1). In total, the sequence was shortened by 13 residues to 270–285 (32) with only a small loss in binding affinity in an FP assay (*K*_D_ = 0.7 μM to *K*_D_ = 2.9 μM).^20^

**Table 1:** The NF-YA derived sequence 267–295 and the six truncated sequences based on this and their binding affinity (*K*_D_) as measured by FP

Nr.	*K*_D_ (μM)	Sequence
2	0.7	_267_VNAKQYHRILKRRQARAKLEAEGKIPKER_295_
33	1.3	_267_VNAKQYHRILKRRQARAKLEAE_288_
34	2.0	_267_VNAKQYHRILKRRQARAKL_285_
35	9.5	_267_VNAKQYHRILKRRQAR_282_
36	2.1	_268_NAKQYHRILKRRQARAKL_285_
32	2.9	_270_KQYHRILKRRQARAKL_285_
37	45	_271_QYHRILKRRQARAKL_285_

Repressor/activator protein 1 (RAP1) is part of the shelterin complex which plays a role in the regulation of telomeres.^78^ The PPI of RAP1 with telomeric repeat-binding factor 2 (TRF2) represses the localisation of poly [ADP-ribose] polymerase 1 (PARP1) to telomeres resulting in catastrophic telomere loss.^79^ The original 41-residue TRF2 interacting sequence, consisting of two α-helices, bound RAP1 with a *K*_D_ of 16.5 nM. This was shortened to a 16-mer peptide (_281_TTIGMMTLKAAFKTLS_296_ (38)) containing only one of the α-helices as a starting point for stapling (see Section 4.1).^80^

Apoptosis-inducing factor (AIF) regulates cellular survival through functions in the mitochondria and AIF mediates neuronal cell death under lethal cellular stress with inhibition of AIF having a neuroprotective effect.^81^ Cyclophilin A (CypA) binding to AIF initiates the translocation of both proteins into the nucleus.^82^ The linear AIF mimicking 25-mer peptide (_370_QSVGVSSGKLLIKLKDGRKVETDHI_394_ (39)) showed binding to CypA (*K*_D_ = 12 μM by SPR) and blocked nuclear translocation.^83^ It was possible for 39 to be shortened by 16 amino acids to the 9-mer section _381_LIKLKDGRKVE_389_ (40) as NMR studies had shown that was the most important section of the interaction.^84^ An increased binding affinity was seen for 40 (*K*_D_ = 2.4 μM) compared to 39 (*K*_D_ = 12 μM by SPR).^85^ Although removal of a large section may not be possible for most peptides, the removal of any residues is still beneficial to improve the drug-like properties of the peptide. The 16-mer peptide (_69_AFFAQLQLDEETGEFL_84_ (23)) derived from Nrf2 was truncated to a 14-mer, 12-mer, 10-mer, 9-mer, 8-mer and 7-mer. From the 16-mer to a 10-mer (_75_QLDEETGEFL_84_ (41)) the binding affinity was maintained (*K*_D_ = 27.3 nM for 41 and *K*_D_ = 23.9 nM for 23). The removal of another residue of the N-terminus to give the 9-mer peptide (_76_LDEETGEFL_84_ (42)) led to a big decrease in binding affinity (*K*_D_ = 352 nM) with any further truncations abolishing binding.^86,87^

## 4. Increasing peptide affinity

Having identified the essential residues, the peptide can be optimised further for binding affinity, cell permeability and proteolytic stability. To increase the binding affinity, it may be possible to introduce mutations into the sequence or use peptide stapling or macrocyclization.^88^ Peptide stapling and/or macrocylisation can also provide an opportunity to increase their cell permeability and proteolytic stability.

### 4.1 Introducing mutations into the sequence

Improving the binding affinity of the designed peptide can be achieved through the introduction of a mutation into the sequence with the possibility to explore unnatural amino acids. It may be possible to increase the size of a hydrophobic residue by introducing substituents or through introducing larger aromatic rings. As described in Section 3.1, an alanine scan may highlight the potential positions at which mutations may be introduced. The PPI between Suppressor of Mothers against Decapentaplegic (Smad) and Yes-associated protein (YAP) is involved in the activation of a signalling pathway leading to heterotopic ossification (HO) characterised by bone formation outside of the skeleton, making it a target for the treatment of HO.^89^ Using computational genetic evolution to sequentially vary the residues within the 205–214 section of Smad an optimised peptide was identified, _205_DGWPPPYPRV_214_ (43) (*K*_D_ = 2.5 μM). An alanine scan of 43 revealed three unimportant residues (G206, P212 and V214) which were computationally varied to all other natural amino acids. Six peptides were explored experimentally with the combination of two mutations (_205_D**Q**WPPPYPR**H**_214_ (44)) leading to a nearly 10-fold improvement in binding (*K*_D_ = 0.34 μM).^90^

**Fig. 11:**
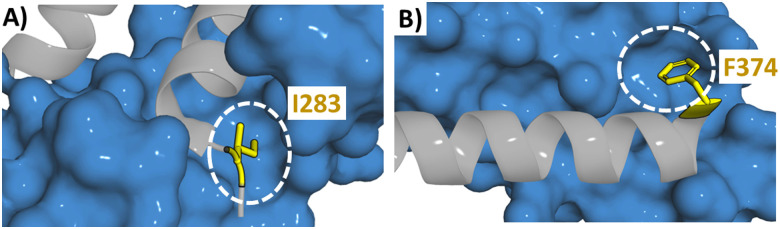
Structure of (A) RAP1 (blue) with TRF2 (grey) (PDB: 3K6G) highlighting the binding pocket of I283 with a white circle and (B) β-catenin (blue) with BCL9 (grey) (PDB: 2GL7) highlighting the binding pocket of F374 with a white circle.

It is not only through an alanine scan that these positions may be highlighted, when the structural information is available, the PPI can be examined. A TRF2 derived peptide (38) has high affinity for RAP1 (*K*_*i*_ = 0.14 μM). The crystal structure of the RAP1/TRF2 complex showed a large hydrophobic space around I283 (Fig. 11A). Therefore different natural and unnatural hydrophobic mutations were attempted at I283 (2,2-dimethylpropylalanine, cyclohexylalanine, phenylalanine, tryptophan, naphthalene and 2, 3 and 4-chlorophenylananine) with the 2-chlorophenylalanine (45) found to increase binding the most (*K*_*i*_ = 7 nM).^80^ Similarly mutations were explored around F374 in a 35-mer linear peptide derived from BCL9 targeting β-catenin (residues 347–381 (46)). The binding pocket of F374 is shallow but wide which could accommodate a larger group (Fig. 11B). Smaller and larger hydrophobic groups were explored with the introduction of a 2-naphthylalanine (47) leading to a 3-fold increase in binding affinity.^91^

A CK2β derived cyclic 13-mer GCRLYGFKIHGCG (9) demonstrated moderate binding affinity for CK2α by ITC (*K*_D_ = 1.75 μM). Docking experiments highlighted *meta*-substitution of F190 with hydrogen or halogen bond forming substituents reached a well-defined water molecule in the structure. Chloro and iodo-substituent analogues were synthesised with the *meta*-iodine (48) resulting in an improved binding affinity (*K*_D_ = 0.239 μM by ITC).^92^

Multiple mutations can also be introduced into a sequence although the combination of individually beneficial mutations may not be additive. A FYCO1 derived 34-mer peptide spanning residues _1275_GQGANTDYRPPDDAVFDIITDEELCQIQESGSSL_1298_ (49) was shown to bind LC3B (*K*_D_ = 0.29 μM) in an FP assay.^93^ The binding pocket of F1280 was shown to be able to accommodate a bigger hydrophobic group and a 2-naphthylalanine substitution (50) increased the binding affinity (*K*_D_ = 0.14 μM). A substitution of L1288 with a *tert*-butylalanine (50) led to a small improvement of binding affinity (*K*_D_ = 0.25 μM). The N-terminal section of the peptide is located near various negatively charged residues of LC3B and an introduction of an Arg to the N-terminus (51) showed a slight improvement of the binding affinity (*K*_D_ = 0.20 μM). A combination of these three mutations (52) led to a peptide with a *K*_D_ of 0.12 μM which was similar to that of the single substituted 50.^94^

### 4.2 Stapling of α-helical peptides

**Fig. 12:**
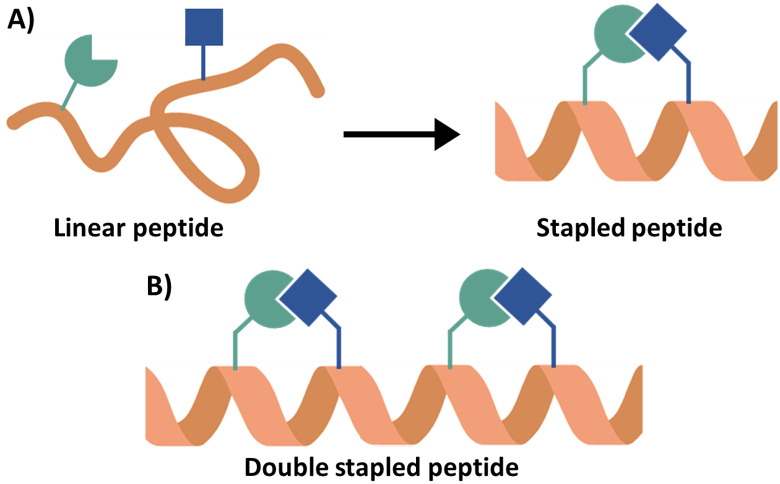
(A) Schematic overview of peptide stapling and (B) schematic overview of a double stapled peptide.

Peptide stapling can increase the binding affinity of a peptide due to the preorganisation of a peptide secondary structure.^95^ In peptide stapling a bond is formed between two residues on the same side of the α-helix (Fig. 12A). This is achieved with canonical amino acids such as cysteines to form thioether stapled peptides, or lysine with aspartic or glutamic acid to create a lactam stapled peptide. Additionally, unnatural amino acids generate commonly used hydrocarbon (formation of an alkene bond *via* ring closing metathesis) and triazole staples. Fairlie and co-workers compared the α-helicity of pentapeptides using six stapling techniques demonstrating that lactam-stapling induced the highest level of α-helicity followed by hydrocarbon stapling.^96^ Hydrocarbon stapling is commonly used due to its ability to increase cell permeability.^97^ Hydrocarbon stapling has been extensively reviewed with many examples of its successful application in increasing binding affinity, proteolytic stability and cell permeability.^23,88,98–100^ Two cases will be highlighted here as examples of the use of a single hydrocarbon staple leading to the peptides with *in vivo* activity.

The PPI between β-catenin and its cofactor B-cell lymphoma 9 (BCL9) is part of the Wnt signalling pathway which plays a key role in cell proliferation with activation of this pathway found in different cancers.^101,102^ From the crystal structure, a 35-mer linear peptide (residues 347–381 (53)) from BCL9 was shown to bind β-catenin with a *K*_D_ of 616 nM in an FP assay. Truncation to a 24-mer (residues 351–375) (54) resulted in a small loss in binding inhibitory effect (*K*_*i*_ = 1.90 μM compared to 0.96 μM) as measured in a competitive FP assay.^103^ This shortened sequence was used in the design of hydrocarbon stapled peptides identifying _351_LSQEQLEHRERSL**S**_**5**_TLR**S**_**5**_IQRMLF_374_ (55) which inhibited the BCL9/β-catenin complex at an IC_50_ of 135 nM. *In vivo* mouse xenograft models of Wnt-driven cancer showed suppression of tumour growth, angiogenesis and invasion with treatment of 55.^104^ Similarly, peptides were derived from Bcl-2-interacting mediator of cell death (BIM) to target antiapoptotic B-cell lymphoma 2 (BCL2) family proteins.^105,106^ The BIM BH3 helix (_146_IWIAQELRRIGDEFNAYYARR_166_ (56)) was modified with *i*, *i* + 4 hydrocarbon stapling at positions 154 and 158, giving peptide 57 (_146_IWIAQELR**S**_**5**_IGD**S**_**5**_FNAYYARR_166_). 57 showed nanomolar binding against a range of BCL-2 family proteins including BCL-X_L_, MCL-1 and BFL-1 and suppressed tumour growth *in vivo*.^107^

Hydrocarbon stapling may however not always prove the best option for structure restraint. In targeting Li-TryR dimer using peptides based on section _435_PEIIQSVGISMKM_447_ (6) it was found that although hydrocarbon stapling gave the most α-helical peptides, only lactam stapled peptides were able to disrupt the dimer formation. Molecular dynamics studies suggested Q439 was not able to adopt the required conformation in the hydrocarbon stapled peptides.^108–110^ Other stapling methods may increase the solubility of the peptides, such when targeting the RAP1/TRF2 PPI in which triazole stapling was applied to peptide 38. The triazole stapled peptide, _281_TTIGMMTLK**Z**AFK**X**LS_296_ (58) was 10-fold more potent than the linear 16-mer peptide 38 (*K*_*i*_ = 0.14 compared to *K*_*i*_ = 2.0 μM in a competitive FP assay). The triazole staple increased the binding affinity (*K*_*i*_ = 0.14 compared to *K*_*i*_ = 2.0 μM) whilst improving its solubility.^80^

Multiple staples can also be introduced into the sequence, which can be beneficial for longer sequences (Fig. 12B). Double hydrocarbon *i*, *i* + 4 stapling was applied to a JAZ9 derived 21-mer peptide (residues 218–238) targeting MYC with both higher α-helicity (45% compared to 25%) and better binding affinity (*K*_D_ = 0.10 μM compared to 0.88 μM and 2.4 μM) seen for the double over the single stapled analogues and inhibition of MYC-related gene expression in *Arabidopsis thaliana* was observed.^111^ The same approach taken in targeting the Rab protein, Rab6a, through deriving a peptide from R6IP,^31^ and for targeting DAP12 homodimerization,^112^ SNARE/synaptotagmin-1^113^ and the p53 PPI with MDM2/MDMX.^114^

### 4.3 Macrocyclisation of peptides

**Fig. 13:**
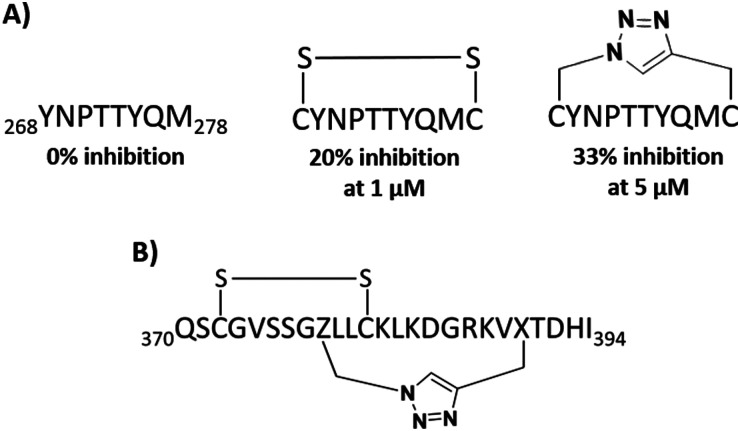
(A) The macrocyclisation of EGFR derived peptide _269_YNPTTYQM_278_ (5) using disulfide (59) and triazole (60) linkers and (B) structure of bicyclic peptide _370_QSCGVSSGZLLCKLKDGRKVXTDHI_394_ (74) derived from AIF.

For α-helical structures stapling can be used to ensure the helical structure of the peptide. For other secondary structures, such as β-hairpin peptides, this can be achieved with macrocylisation. The EGFR dimerization arm residues _269_YNPTTYQM_278_ (5) form a β-hairpin with a cyclic version of this peptide, CYNPTTYQMC (59), decreasing dimer formation by 20% at 1 μM (Fig. 13A).^33^ A triazole cyclised version (60) inhibited EGFR dimerization by 33% at 5 μM, similar to previous disulfide cyclised peptides. However, the proteolytic stability was improved for the triazolyl-cyclised peptide over the disulfide version.^115^

Vestigial-like protein (VGLL) is a coactivator of transcriptional enhanced associate domains (TEADs) and has been found to be a transcriptional repressor inhibiting tumorigenesis caused by YAP as part of the Hippo pathway with VGLL4 competing with YAP for binding to TEADs.^116^ VGLL4 is a small protein consisting of an α-helix linked to a double-stranded β-sheet through a loop linker. To mimic the double-stranded β-sheet cysteine residues were added to the peptide termini to allow for head-to-tail cyclisation across residues _19_PKTEWNAGSVIFTY_32_ and this sequence was conjugated to the cell penetrating peptide TAT (see Section 5 for more information) to give peptide 61. 61 showed increased activity in a cell viability assay (IC_50_ = 18.1 μM) compared to its linear counterpart (IC_50_ = 87.4 μM) and the slightly shorter cyclised _20_KTEWNAGSVIFT_31_ (62) showed a similar activity (IC_50_ = 25.0 μM) with its linear counterpart showing negligible activity (IC_50_ > 100 μM).^117^

The linear CK2β peptide _186_RLYGFKIH_193_ (8) forms a β-hairpin loop with Y188 at the turn (see Section 2.2). The peptide was extended and Cys residues were added which facilitated cyclisation through a disulfide bridge giving the 13-mer peptide GCRLYGFKIHGCG (9). This peptide inhibited the CK2α/CK2β complex with an IC_50_ of 3 μM, a ten-fold increase on the linear version (IC_50_ = 30 μM).^45^ The replacement of the disulfide bridge with a triazole based bridge further increased binding (*K*_D_ = 460 nM) compared to 9 (*K*_D_ = 1000 nM) as measured by ITC.^118^

The linear AIF mimicking 25-mer peptide (370–394) (39) showed binding to CypA and blocked nuclear translocation.^83^ This section forms a β-hairpin and so disulfide and triazole cyclisation were applied to create mono and bicyclic peptides. A peptide with a combination of disulfide and a triazole bridge, _370_QSCGVSSGZLLCKLKDGRKVXTDHI_394_ (63) (Fig. 13B) showed improved binding (*K*_D_ = 0.85 μM) compared to the 39 (*K*_D_ = 5.0 μM).^119^

Macrocyclisation strategies for β-hairpins as well as helix-turn-helix peptides have been recently reviewed.^88^

## 5. Increasing cell permeability

Key for the effectiveness of drugs with intracellular targets is their ability to cross the cell membrane. The main structure of a cell membrane is made up of a lipid bilayer formed by phospholipids, containing a hydrophilic head and hydrophobic tail. The bilayer is formed with the hydrophilic heads on the outside and the hydrophobic tails on the inside.^120^ Therefore for the passive absorption of drugs, they have to pass through this hydrophobic interior. The development of peptide therapeutics is often hindered by their lack of cell permeability due to the hydrophilic character of the backbone amide bonds as well as the side chains of some amino acids. Hydrocarbon stapling commonly increases the lipophilicity of peptides and can increase cell permeability. However, it may not be possible to insert a staple into a peptide sequence, especially in the development of non α-helical peptides. It has also been found that cyclic peptides may have enhanced cell permeability due to their conformation where the hydrogen bonds are formed within the peptide, leaving a hydrophobic exterior.^121^ Other methods include the methylation of the backbone nitrogen, removing its ability for hydrogen bonding.^122,123^ However, the increasing lipophilicity is not the only option in promoting cell permeability. Cell-penetrating peptides (CPPs) have been developed which can be conjugated to the main peptide, with this topic having been reviewed extensively.^124–127^ Many different CPPs have now been developed, but a similarity between them all is their highly positively charged nature. It is thought that their uptake may be not *via* passive absorption but through endocytosis or by direct translocation. Endocytosis is a process in which the peptide is surrounded by the hydrophilic outside of the cell membrane and then taken into the cell forming a vesicle with the peptide inside. In direct translocation the binding of the positively charged peptide to the negatively charged membrane causes instability in the membrane. This causes pore formation through which the peptide can enter the cell. This benefit may come at a cost, with toxicity sometimes observed at therapeutically relevant concentrations. Despite some conjugates progressing to phase III trials, no CPP containing peptides have progressed to clinical use.

The conjugation of a CPP to a potent peptide to enhance cell permeability was successfully applied to the disulfide cyclised YAP derived peptide. Although the peptide showed high binding affinity (IC_50_ = 15 nM in competitive SPR), it was not able to penetrate cells. Therefore, the peptide was conjugated to TAT, a CPP derived from HIV, with cell permeability now seen.^75,76^ Alternatively, the addition of two or three arginine residues to the end of the peptide has also been shown to increase cell permeability by changing the overall charge without the need to add a large sequence of amino acids. For example, a hydrocarbon stapled peptide based on peptide 1 derived from SOS1 required the addition of two Arg residues, changing the overall charge from −1 to +1, for cellular uptake.^18^

Instead of improving the cell permeability of the peptides through the addition of a CPP or arginine residues, mutations can be introduced to change the overall charge. A double hydrocarbon-stapled R6IP derived peptide targeting Rab6a (_900_DDE**S**_**5**_EWF**S**_**5**_YHL**S**_**5**_FFN**S**_**5**_V_916_ (64)) demonstrated a *K*_D_ of 7.8 μM, but cellular uptake was limited. Introduction of D901N and E904Q (65) removed the negative charges and increased cellular uptake (cellular uptake in range of TAT) with only a small loss in binding affinity (*K*_D_ = 12.7 μM).^128^ This method was also applied to a p53 peptide (_17_ETFSDLWKLLPE_28_ (66)). Through a double staple of hydrocarbon stapling at positions 17 and 21 and lactam stapling at positions 24 and 28 as well as mutations L22K and P27R, giving peptide 67 (Ac-S_5_TFSS_5_KWDLLRK-NH_2_), the overall charge was changed from −2 to +2. This peptide reduced cell viability whereas its counterpart without mutations (giving an overall neutral charge) did not affect cell viability.^114^

## 6. Conclusion and future outlook

Peptides for the inhibition of protein–protein interactions offer several advantages over antibodies and small molecules. Peptides are inexpensive, structurally designed, and readily modified, while offering exquisite selectivity for their target. Peptides cover large protein interaction surfaces and have demonstrated high levels of success for the inhibition of biomacromolecule interactions. We have highlighted the conceptually elegant and simple method of deriving a peptide to inhibit a PPI from one of the interacting protein partners. Using the information available about the interaction interface an initial sequence for investigation can be chosen. High amounts of structural information allow for the extraction of secondary motifs from the interface. With limited structural information available the interaction between the proteins can be analysed using protein mutagenesis to understand the importance of regions or residues for the interactions. When only sequence information is available peptide arrays allow for the synthesis of a large library of peptides which can span large sections of protein an identify binding motifs. Similarly, conserved regions across different proteins or species indicate the importance of that section of the sequence and offer an excellent initial sequence. After a binding sequence has been identified the sequence can be analysed using an alanine scan to provide information about the key residues for the interaction and the sequence may be shortened to improve the drug-like properties of the peptide. To further increase the binding affinity of the selected peptides mutations may be introduced or conformational constraints (peptide stapling or macrocyclization) may be applied. Further modifications can be made if necessary to improve the cellular uptake by increasing the positive charge of the peptide or by conjugation to a cell-penetrating peptide. These methods have resulted in many examples of peptide inhibitors for a wide array of interactions.

Recent developments in the identification of protein–protein interactions, and with structural information increasingly available through advances in protein structure prediction and cryo-EM, this method of identifying peptide inhibitors for biomacromolecular interactions has become more accessible and can allow for the exploration of previously unexplored interactions including protein–protein, protein–DNA, and protein–RNA interactions.

## Data availability

All data is presented in the manuscript or the appropriate citation.

## Conflicts of interest

There are no conflicts to declare.
